# Pooling of Wealth in Marriage: The Role of Premarital Cohabitation

**DOI:** 10.1007/s10680-022-09627-2

**Published:** 2022-08-09

**Authors:** Agnese Vitali, Romina Fraboni

**Affiliations:** 1 Department of Sociology and Social Research, University of Trento, Trento, Italy; 2 Istat‐Italian National Institute of Statistics, Rome, Italy

**Keywords:** Cohabitation, Community of property, Marital property, Marriage, Resource pooling, Separation of Property

## Abstract

Previous studies documented the existence of a ‘cohabitation–marriage gap’ in resource pooling among opposite-sex partners, with cohabiters being more likely to separate income and wealth than married individuals. Surprisingly, despite many non-marital cohabitations transform into marriages, we know little about income and wealth pooling of ‘spousal cohabiters’, i.e. spouses who transition to marriage after experiencing a period of non-marital cohabitation. The comparison between ‘spousal cohabiters’ and directly married spouses is particularly interesting because it offers a litmus test of theories of marriage in relation to how and why economic resources are differently distributed within married vs. cohabiting couples. This paper compares directly married couples and ‘spousal cohabiters’ in Italy, focusing on one aspect of resource pooling: the marital property regime, i.e. the choice made at the time of marriage between joint or separate ownership of wealth accumulated during marriage. Competing hypotheses are developed on the basis of the arguments that marriage yields legal protection, that selection mechanisms drive both the choice of community vs. separation of property and direct marriage vs. premarital cohabitation, and that, by inertia, ‘spousal cohabiters’ continue to separate resources upon transition to marriage. Results based on the 2016 Italian ‘Family and social subjects’ survey show that ‘spousal cohabiters’ are significantly more likely to choose separation of property compared to directly married spouses. Such differences, however, are drastically reduced once relevant confounders are controlled for, hence suggesting that existing differences between directly married and previously cohabiting couples and, more generally, differences between married and cohabiting couples are driven, above all, by selection mechanisms.

**Supplementary Information:**

The online version contains supplementary material available at 10.1007/s10680-022-09627-2.

## Introduction

For many, cohabitation is nowadays the preferred way to enter a co-residential partnership, and transition to parenthood among cohabiters has become increasingly common in Europe, including in Italy. Accordingly, a growing literature has been researching whether cohabiting partners manage their economic resources and share wealth differently than married spouses. Empirical results suggest that cohabiting couples are more likely to keep their resources separate than married couples (Evans & Gray, 2021; Hamplova & Bourdais, 2009; Heimdal & Houseknecht, 2003; Lyngstad et al., 2011; Vogler, 2005; Vogler et al., 2008; Winkler, 1997).

Past research focused mainly on income, while wealth within couples has been less researched. Obviously, income and wealth are intrinsically different, and the underlying reasons driving couples to pool their income and wealth can be different too, as well as the possible consequences of pooling income and wealth (see Lersch et al. 2022 in this special issue). The mechanisms driving couples to pool their economic resources (e.g. towards a joint purchase or investment decisions, or the choice of one allocative system) and to manage income in everyday practices are also different. Nonetheless, the above-mentioned existing studies of income pooling, wealth pooling, and income management among partners tend to agree that a ‘cohabitation–marriage gap’ exists in that cohabiters are more likely than married individuals to keep and manage at least part of their incomes and wealth separately. For the sake of simplicity, in the following, we refer to ‘resource pooling’ for identifying studies of income management, income pooling, and wealth pooling.

Even if non-marital cohabitation is now widespread, marriage is not foregone—especially in Italy—rather, it is preceded by one or more experiences of premarital cohabitation. Despite the fact that a growing number of recent marriages are initiated as non-marital cohabitations, little knowledge exists on the management and pooling of economic resources in general, and of wealth in particular, for ‘spousal cohabiters’ (Vespa & Painter, 2011), i.e. spouses who cohabited together before marriage, with the exception of Hiekel et al. (2014). The scant attention to whether and how cohabiters change their resource management and pooling strategies upon marriage, and more generally the scant attention to the comparison between directly married and previously cohabiting spouses, are surprising because these comparisons may contribute to unravel the ‘cohabitation–marriage gap’ in resource management and pooling.

Are cohabiting couples who transition to marriage more, less, or equally likely to pool incomes and wealth at marriage compared to directly married spouses? Are spouses with a prior experience of cohabitation more likely to retain separate resources upon marriage, thus behaving more similarly to cohabiters, even after they transition to marriage? Or will they behave more similarly to married couples? The answers to these questions can inform us about the validity of theories of marriage developed to explain why cohabiting partners are less inclined to pool incomes and wealth than married spouses.

Take for instance the explanations for the ‘cohabitation–marriage gap’ based on legal protection, social norms, and selections mechanisms. With respect to the legal protection argument, if no difference was to be found in resource pooling between previously cohabiting couples and directly married couples, we would conclude that the known lower preference for resource pooling among cohabiting couples compared to married couples is essentially due to their lower legal protection. In other words, marriage would offer the legal base for justifying joint investments. According to the social norm argument, the institution of marriage would provide a set of social norms regulating how resources shall be distributed among partners—social norms which are lacking for cohabiting couples (Eickmeyer et al., 2019; Nock, 1995). With respect to selection effects, the literature suggests that the preference for separate resources may not be linked to (previous) cohabitation vs. marriage. Rather, it may be linked to different socio-demographic and economic characteristics among the two couple types (Lyngstad et al., 2011; Treas, 1993). Evidence of selection mechanisms is found if, after relevant confounders are appropriately controlled for, differences in resource pooling among the two groups are reduced or disappear. If, instead, previously cohabiting couples were more likely to continue keeping resources separate after marriage, even after all relevant confounder are controlled for, we would disprove the legal protection and the social norms argument and cast doubt on the selection effects argument. In this case, we would find support for what we call the ‘inertia’ hypothesis, according to which ‘spousal cohabiters’ become accustomed to separate at least some of their economic resources during the period of non-marital cohabitation and continue to separate resources upon transition to marriage.

Comparing directly married and previously cohabiting spouses hence offers a litmus test of theories of marriage in relation to how economic resources are distributed, owned, and managed by partners in married vs. cohabiting couples. Because they transition from non-marital cohabitation to marriage, ‘spousal cohabiters’ allow a better test of theories of marriage than comparing cohabiting versus married individuals as done in previous research. This paper applies a litmus test using a measure of wealth ownership among spouses and extends previous knowledge on how married couples organize their economic resources by investigating whether the intra-couple allocation of wealth is associated with the experience of premarital cohabitation.

Our study is based on Italy, for which we know very little not only in terms of wealth pooling, but also in terms of income pooling between partners, compared to other countries. Most previous research on income management and pooling among partners is based on survey questions contained in the module ‘Family and Changing Gender Roles’ of the International Social Survey Programme fielded in 1994, 2002, and 2012. However, such relevant questions are only available for the Italian sample in 1994. For this reason, previous research on Italy is based on the choice of the marital property regime (Barbagli, 1993, 1997; Fraboni & Vitali, 2019) or household headship as a measure of responsibility for financial and economic choices (Bertocchi et al., 2014).

We too use the marital property regime, a measure of resource pooling among spouses governing whether the couple’s wealth accumulated during marriage is shared equally among spouses or kept separate. A similar arrangement does not exist for cohabiting couples. The marital property regime is a legally binding choice made jointly by the spouses at the time of marriage, a choice that will determine, among other things, the distribution of their assets in case of divorce. All married couples in Italy need to choose, at the time of marriage (i.e. when they sign the marital certificate), between the community or the separation of property. A detailed description of the marital property regime in Italy, its history and evolution, and the consequences of choosing community vs. separation of property can be found in Fraboni and Vitali (2019). Analyses are based on the 2016 Italian multipurpose survey, that collects rich information on the characteristics of spouses at marriage, and it enables the consideration of many key confounding effects identified in previous literature (religiosity, education, presence of own vs. children in common, duration of relationship).

Results show that previously cohabiting spouses are more likely to opt for the separation of property at marriage, hence suggesting the existence of a (premarital) ‘cohabitation–marriage gap’ in wealth ownership in Italy, as is the case in many other countries. Results however also show that once relevant confounders are accounted for, the experience of cohabitation per se is no longer associated with separation of marital property. Rather, several socio-demographic and economic characteristics drive couples both into premarital cohabitation and separation of property, hence suggesting that selection mechanisms are the main explanation for the (premarital) ‘cohabitation–marriage gap’ in wealth ownership (and possibly also in other measures of resource pooling), at least in Italy.

## Premarital Cohabitation in Italy

Italy has been a laggard in the uptake of the Second Demographic Transition. ‘New’ partnership behaviours, such as premarital cohabitation, have taken longer to diffuse in Italy compared to other European countries (Di Giulio & Rosina, 2007; Nazio & Blossfeld, 2003; Rosina & Fraboni, 2004).

And yet, contrary to what many commentators think, there was a time when premarital cohabitation was common in parts of Italy, especially in the South, among the lower socio-economic strata and in rural contexts (Sabbadini, 1991). This type of non-marital cohabitation was short-lived, involving the couple sleeping away from the respective parental homes for a few days up to a few months. Sometimes such practice was consensual: when a couple wanted to marry but were opposed by the parents, the lovers would organize an escape, called ‘fuitina’ in Sicilian dialect. Sometimes, however it was not consensual, and could involve a rape. Generally, in both cases, the event would be followed by marriage to ‘repair’ the woman’s honour, and forgive the man. The practice was recognized by the legal system: until as late as 1981, men who kidnapped women were absolved if, after the kidnap, the woman agreed to marry the man, even if the kidnapping was non-consensual. ‘Fuitina’ lost ground over time and virtually disappeared (Vitale, 2020).

With the exception of the kidnapping practice, confined to Southern Italy, modern cohabitation remained marginal in Italy through the 2000s (De Rose et al., 2008), when it was already widespread in Western Europe. Women born in the early 1970s were only marginally more likely to have ever experienced cohabitation compared to women born in the 1950s and 1960s (Nazio & Blossfeld, 2003). Current research on Italy suggests that, at least until the 2010s, cohabitation was considered unconventional in Italy and its initial pattern of diffusion conforms to the idea of a Second Demographic Transition, i.e. to the spread of new values and ideas of individualization, freedom, and secularization (Barbagli et al., 2003; Guetto et al., 2016; Nazio & Blossfeld, 2003). The slow diffusion of non-marital cohabitation originated in the Northern regions and among the higher educated (Aassve et al., 2015; Di Giulio & Rosina, 2007; Rosina & Fraboni, 2004). Non-marital childbearing was considered even more unconventional. Perelli-Harris (2014) estimated that in Italy only 5% of first births occurred to cohabiting mothers between 1985 and 2000. Births out of wedlock have then risen from 7.4% in 1993 to 33.4% in 2019 (source: own analysis of live births registrations and Istat, 2020).

For Italy, there is also support for a pattern of disadvantage (Perelli-Harris, 2010), i.e. for the idea that cohabitation may be linked to poor economic- and employment-related prospects. Non-marital cohabitation was found to be associated with partners’ employment uncertainty and economic characteristics (Salvini, 2015; Vignoli et al., 2016). For some, cohabitation is therefore preferred to marriage for its lower level of commitment during periods of own economic uncertainty and therefore could be a holding pattern to marriage, until economic and employment stability is reached. Furthermore, some Italian cohabiters reported among the benefits of cohabitation vs. marriage the lack of costs linked to divorce, in terms of money and time needed to engage with the legal system (Vignoli & Salvini, 2014).

## The Marital Property Regime in Italy

We study a measure of wealth ownership linked to a choice made by newly wed spouses at the time of marriage, i.e. their chosen marital property regime. This choice legally regulates how spouses will manage and administer the wealth accumulated during the course of their marriage (properties, including rental income from properties; vehicles; and revenue from business). The choice is between community or separation of property and encompasses all assets accumulated by either spouse since the time of marriage. The marital property regime chosen by the couple at the time of marriage also regulates how their wealth will be distributed in the eventuality of divorce, i.e. if the couple chose the community of property, all assets accumulated during marriage are jointly owned, hence shared upon divorce, independently of whether one or both spouses have contributed to pay for purchasing such assets. Instead, the payment of alimony upon divorce is independent of the type of property regime chosen. Negative assets are shared under the community but not the separation of property. For this reason, separation is generally preferred by couples with at least a self-employed spouse so to protect the assets of the other spouse in case on bankruptcy (Fraboni & Vitali, 2019).

The community of property hence protects the lower-status spouse by granting access to half of the assets on divorce. Under the separation of property, instead, each spouse is, and remains on divorce, the sole owner of the assets (s)he purchased. The chosen marital property regime, hence, has long-lasting economic effects which extend beyond the duration of the marital union on divorce. Also, income pooling can be easily reversed, e.g. when employment-related circumstances of either partner change, possibly through the initiative of one spouse, whereas the choice of the marital property regime can only be changed upon agreement of both spouses in front of a notary, with payment of a fee.

The marital property regime is an institution governing the distribution of assets among married partners. For this reason, we do not consider lifelong cohabiters who may, if they wish, sign a contract governing the ownership of their assets, as two unrelated persons may do. We suspect few of them would do so, given that such a contract is expensive and it clashes with most meanings attached to cohabitation in Italy: if cohabitation is a test of the relationship (Perelli-Harris et al., 2014), it does not make sense to legally regulate its economic arrangements; if cohabitation is itself a protection in the eventuality of divorce (Vignoli & Salvini, 2014), cohabiters have no incentive to regulate their property; if cohabitation is a waiting pattern or an alternative to marriage due to scarce economic resources (Salvini, 2015; Vignoli, et al., 2016), hardly any cohabiters would pay to regulate their property (Wilmoth & Koso, 2002).

## Background and Hypotheses

### Pooling of Economic Resources: Marriage Versus Cohabitation

Previous studies generally found that cohabiting couples are more likely than married couples to separate, at least partly, their economic resources (Winkler, 1997; Heimdal & Houseknecht, 2003; Vogler, 2005; Vogler et al., 2008; Hamplova & Bourdais, 2009; Lyngstad et al., 2011; Evans & Gray, 2021).

Most existing research is based on the organization of household income, e.g. via the choice of a given allocative system whereby one partner manages most or all incomes, or partners pool completely or partly their incomes, frequently based on Pahl’s (1989) typology (Evans & Gray, 2021; Hamplova & Bourdais, 2009; Heimdal & Houseknecht, 2003; Kenney, 2004; Vogler et al., 2008). Some studies are based on the use of shared bank accounts, household expenses (Addo, 2017; Lyngstad et al., 2011), and credit cards (Addo, 2017). Furthermore, most previous research is based on data from the International Social Survey Programme (ISSP). For Italy, ISSP-related questions on income sharing among partners are available only for the year 1994, hence, for Italy, we know little with respect to income pooling.

Less is known about differences in wealth ownership between married and cohabiting couples, with few exceptions. Addo (2017) using American cohort data, finds that cohabiting couples are less likely to share homeownership, including sharing a mortgage, compared to married couples. Holland (2012) finds for Sweden that married couples and cohabiters who intend to marry are more likely to share homeownership, and suggests that marriage may be a prerequisite for buying a property together. Leturcq & Frémeaux 2022 in this special issue find that cohabiting couples hold less housing wealth and less financial wealth compared to married couples; however, over time, they accumulate housing wealth at a similar pace, and accumulate financial wealth at a faster pace than their married counterparts. Kan and Laurie (2014) find that cohabiters in the UK are less likely to share savings, investments, and debts than married individuals.

The literature generally explains the observed differences between married and cohabiting individuals in pooling economic resources, whether income or wealth, on the basis of (1) selection mechanisms; (2) marriage as a social institution; (3) different legal protection granted to marriage and cohabitation.

Differences in resource pooling among married and cohabiting couples may depend on the fact that partners self-select into marriage or cohabitation on the basis of certain characteristics, which in turn are associated with the preference for separation or pooling of economic resources. Therefore, controlling for those characteristics is crucial to understand the ‘cohabitation–marriage gap’, i.e. in evaluating to what extent the different management and pooling of economic resources are due to the type of union, and to what extent it is due to confounding factors (Hiekel et al., 2014; Lyngstad et al., 2011). It is therefore useful to review existing findings on the correlates of pooling of economic resources among married and cohabiting couples. Italian cohabiters are more often dual earners and educationally homogamous than married individuals, more highly educated, more likely to live in urban contexts, more likely to be childless and with shorter union histories compared to married spouses (De Rose & Fraboni, 2015; Di Giulio & Rosina, 2007; Guetto et al., 2016; Rosina & Fraboni, 2004). Cohabiters also tend to be less religious and less traditional (Clarkberg et al., 1995); for example, in Italy they are less likely to meet their mother in person on a daily basis compared to married individuals (Pirani, 2020). Previous international research showed that individuals with such characteristics are more likely to keep their economic resources separate (Hiekel et al., 2014; Treas, 1993) and also more likely to choose separation of property at marriage (Fraboni & Vitali, 2019). Religiosity and traditionalism are associated both with direct marriage and with pooling of economic resources (Pahl, 1989). Similarly, individualism, autonomy and independence, i.e. post-materialistic values, are typical of people who experience cohabitation, as suggested by the Second Demographic Transition (Lesthaeghe, 2010) and may be negatively associated with pooling. Previous research agrees that separation of resources is especially widespread among childless cohabiters (Vogler et al., 2008). Having a child together, instead, is associated with resource pooling among cohabiting and married couples alike: a child in common is in fact a sign of commitment among partners (Kenney, 2004; Vogler et al., 2008; Winkler, 1997). On the contrary, having a child from a previous relationship is associated with at least a partial separation of economic resources (Kenney, 2004), particularly among married couples (Eickmeyer et al., 2019). The longer the relationship, the more likely cohabiters are to pool their economic resources (Lyngstad et al., 2011; Winkler, 1997). Resource pooling is less likely when at least one partner was previously married (Heimdal & Houseknecht, 2003; Treas, 1993) or experienced the dissolution of a previous non-marital cohabitation (Vogler et al., 2008). The association between absolute economic resources and income pooling, instead, is not clear cut. Treas (1993) finds mixed results regarding household income. Hiekel et al. (2014) find that higher education, female employment, and both partners being employed are associated with income separation. Evans and Gray (2021) find evidence that a pattern of disadvantage is associated with resource pooling among cohabiters and married individuals in countries where cohabitation is widespread, especially among low-income couples, whereas it is associated with separation in other countries. Finally, pooling economic resources is associated with partners’ relative economic characteristics such as income and education: in couples where men are economically advantaged compared to women, pooling is more likely, while the opposite is true in couples with economically advantaged women (Brines & Joyner, 1999; Fraboni & Vitali, 2019; Lott, 2017).

Another reason why married and cohabiting couples manage economic resources differently is linked to the very nature of their union (Brines & Joyner, 1999; Hamplova & Bourdais, 2009; Hiekel et al., 2014; Lyngstad et al., 2011). Cohabitation, being an ‘incomplete institution’, lacks prescribed norms guiding how resources should be shared among partners, differently from marriage (Eickmeyer et al., 2019; Nock, 1995). Marriage represents a greater financial and social commitment than cohabitation (Holland, 2013). Married spouses would be more committed since they publicly make a promise to each other, a promise which is costlier to break, in both economic and social terms, and which brings greater social expectations of reciprocity among partners, compared to cohabiting couples. Commitment, in turn, justifies pooling of resources with the aim of minimizing transaction costs (Treas, 1993), leading to a more efficient relationship. Conversely, lower commitment and the uncertainty about the outcome of cohabitation would make resource pooling riskier among cohabiters, and separation of resources more efficient (Belleau et al., 2017; Treas, 1993). However, those cohabiters who are committed, e.g. because they intend to marry, have a child in common, or do not plan to separate from their current partner, manage their economic resources more similarly to married spouses than to non-committed cohabiters (Hiekel et al., 2014; Holland, 2012; Lyngstad et al., 2011). In other words, after possible selection mechanisms are controlled for, when non-marital cohabitations are similar to marriages, and in particular when there are children in common and long-lasting unions, the ‘cohabitation–marriage gap’ in resource management and pooling reduces. On the basis of these empirical results, commentators concluded that the ‘cohabitation–marriage gap’ must be due to the intrinsically different nature of marriage and cohabitation (Hiekel et al., 2014; Lyngstad et al., 2011).

Another explanation for the different management and pooling of economic resources between married and cohabiting couples is linked to their legal status. Cohabitation in several countries—and certainly so in Italy—is characterized by little or no legal protection (Perelli-Harris & Gassen, 2012). Unmarried partners are not legally responsible for one another, and their economic resources are not protected in the eventuality of union dissolution (Wilmoth & Koso, 2002). For this reason, cohabiting partners would be less inclined to pool their resources and poll their wealth than married spouses, even though many may be ignorant of the legal rules that apply to unmarried couples in the eventuality of break-up (Belleau et al., 2017). For instance, in Italy, and many other European countries, cohabiting partners have no rights to alimony payments after union break-up; in a handful of countries they have some rights, though fewer than married partners, and only in Slovenia cohabiters have the same rights than married spouses (Miho & Thévenon, 2020).

Finally, taxation systems based on individual- vs. (married) couple-based income tax as, e.g. in Germany (but not in Italy) can also incentivize pooling of wealth among married but not cohabiting couples (Evans & Gray, 2021; Kapelle et al. 2022 in this special issue).

We acknowledge that earlier literature cited in this paper spans a period during which the prevalence of cohabitation has changed, and that it does not always distinguish between cohabitation as a prelude or alternative to marriage—factors which may help to unravel the ‘cohabitation–marriage gap’. Finally, we acknowledge that earlier research has found mixed results across countries regarding differences between married and cohabiting partners. For instance, Pepin and Cohen (2021) find that the ‘cohabitation–marriage’ gap in income pooling and management is higher in countries with low gender equality. We may therefore expect the cohabitation–marriage gap to be quite wide in Italy, a context that scores generally low in international classifications of gender equality. For what concerns the comparison between the directly married and those with a previous experience of cohabitation, instead, research is scant, also due to the fact that information on premarital cohabitation is unfrequently collected in social surveys.

### Pooling of Economic Resources: Direct Marriage vs. Marriage After Premarital Cohabitation

While the link between cohabitation and separation of economic resources has been established by existing research, we do not know much about the management and pooling of economic resources among married spouses with a prior experience of cohabitation. And yet, premarital cohabitation is increasingly widespread: most marriages nowadays are preceded by ‘spousal cohabitation’. Studying the behaviour of married, previously cohabiting spouses, and comparing it to the behaviour of directly married spouses, we can shed light on the role of marriage for resource pooling. Studying the behaviour of spousal cohabiters allows to go one step further to simply compare cohabiting versus married individuals as done in previous research, and see whether theories of marriage in relation to how economic resources are distributed holds once cohabiters transition to marriage.

For a start, it is useful to understand if and how ‘spousal cohabiters’ differ from spouses who married directly and if and how they differ from cohabiters who do not transition to marriage. From previous (scant) research we know that cohabiters who intend to marry behave more similarly to married spouses than to cohabiters with no marriage plans in terms of income pooling (Hiekel et al., 2014; Holland, 2012; Lyngstad et al., 2011). Pooling may actually be a way to prepare for married life during premarital cohabitation for those who intend to marry (Addo, 2017). This premarital pooling among cohabiters may grant them an economic advantage over the directly married, in the long run. Cohabiters generally have lower wealth than married couples (Lersch, 2017; Leturcq & Frémeaux 2022 in this special issue), particularly in terms of housing wealth (Kapelle & Lersch, 2020). Spousal cohabiters, despite owning less wealth than the directly married at the time of marriage, over time accumulate more wealth and hence, in the long run, married couples with a one-and-only prior experience of non-marital cohabitation enjoy a wealth premium compared to the directly married (Vespa & Painter, 2011). Cohabiters may change their income- and wealth-pooling strategies patterns once they marry, hence becoming more similar to directly married spouses (Burgoyne et al., 2007; Lott, 2017). This may happen because marriage yields a promise of long-term commitment which is better suited for pooling, compared to non-marital cohabitation. We can therefore expect that marriage gives the institutional and legal protection needed for joint investments and is therefore the most important trigger for pooling economic resources. Hiekel et al. (2014) find partial support for this expectation. Their results show that, once relevant confounders are accounted for, spousal cohabiters are equally likely to separate their economic resources as directly married spouses in four countries (Germany, Bulgaria, Georgia, and Russia) out of six (pooling is less likely in Romania and more likely in France). We can speculate that Italy may behave similarly to Germany, of all countries included in their sample, due to similar family patterns and level of gender equality. Fulda and Lersch (2018) find that marriage in itself does not increase the time horizon to which cohabiters plan their saving and spending: transitioning from cohabitation to marriage does not change partners’ financial planning horizons, at least in a country context, Australia, which grants similar legal protection to marriage and non-marital cohabitation.

Finally, it is useful to understand if and how ‘spousal cohabiters’ differ from cohabiters who do not transition to marriage. In other words, it is useful to understand under what conditions cohabiters generally transition to marriage. The American literature suggests that cohabiters typically transition to marriage after reaching a certain ‘economic bar’ (Ishizuka, 2018), when they have good socio-economic prospects, especially for men (Oppenheimer, 2003; Smock & Manning, 1997), and when they have accumulated wealth in the form of owning a vehicle, financial assets, and other assets, again especially for men (Schneider, 2011). These patterns are confirmed for Italy: qualitative studies on the meaning of cohabitation and marriage among Italian couples highlight the importance of economic stability for the transition from cohabitation to marriage (Vignoli & Salvini, 2014). In addition, Italian cohabiters are found to transition to marriage: when they perceive social pressure, especially from the family of origin; pushed by an entrenched will to preserve traditions, also linked to the wedding ceremony; and to have access to legal privileges (Salvini, 2015; Vignoli et al., 2016). Barbagli et al. (2003) found that cohabiters who transitioned to marriage in the 1980s and 1990s were less likely to hold religious and sumptuous wedding ceremonies. For instance, they spent less, on average, on the wedding ceremony, were less likely to hold a banquet and go on honeymoon, and their parents were less likely to contribute to wedding-related expenses.

The literature reviewed in the previous section identified three main mechanisms that can explain why cohabiting and directly married couples have different income pooling strategies: (1) marriage as a social institution; (2) selection mechanism; (3) different legal protection. How do these mechanisms apply to the comparison between directly married and previously cohabiting couples and on the choice between separation and community of marital property?

There are two important considerations suggesting that no difference in pooling should be expected between premarital cohabitation and direct marriage. First, the explanation that marriage and cohabitation have a different nature, with marriage being a social institution and cohabitation being an ‘incomplete’ institution, does not apply in this case, given that both couple types have chosen to transition to marriage. Second, if selection mechanisms are in place, and are causing different pooling strategies between the two couple types, we shall expect these differences to disappear once relevant confounders are controlled for. Hence, once controlling for individual- and couple-specific characteristics, including the two that previous studies identified as the most important for explaining the ‘cohabitation–marriage gap’ in income pooling, i.e. union duration and presence of children (Hiekel et al., 2014; Lyngstad et al., 2011; Winkler, 1997), we would expect experience of premarital cohabitation not to be associated with the choice of property regime. We therefore formulate a first hypothesis:

#### H1 (Selection)

 Couples with a prior experience of cohabitation will be more likely to opt for separation (vs. community) of property at marriage compared to directly married couples. However, once relevant confounders are accounted for, couples will be equally likely to opt for separation or community of property, independently of whether or not they had a prior experience of cohabitation. 

According to H1, we would expect to find significant differences between the two couple types in the null model, where no control variables are included, but such differences shall become negligible once control variables are included in the full model.

If, instead, the different degree of legal protection between marriage and cohabitation is responsible for the different management and pooling of economic resources between the two couple types observed by previous literature, we shall expect no difference between directly married and previously cohabiting couples once they transition to marriage, as marriage grants the rights and institutionally protected base for joint investments. Therefore, we formulate a competing hypothesis:

#### H2 (Legal protection)

Couples with a prior experience of cohabitation will be equally likely to opt for separation (vs. community) of property at marriage compared to directly married couples, independently of whether confounders are controlled for or not.

According to H2, we would expect to find no difference between the two couple types, both in the null model and in the full model with control variables.

However, one additional mechanism may be in place: inertia. Partners who have a premarital experience of cohabitation have experience of managing economic resources while co-residing with their partner, e.g. via the management of a property, ranging from paying a deposit, mortgage, rent or simply running expenses like household bills. They may have experience with buying other assets, such as a car. They further have experience with the management of their individual incomes. The research reviewed above shows that most cohabiters generally keep and manage their money separately. The habit of keeping at least some of their resources for themselves may translate in a higher likelihood of choosing the separation rather than the community of property when they transition to marriage, compared to directly married individuals who do not have experience with managing money and assets while being in a co-residing union. Furthermore, in the specific case of Italy, cohabitation is frequently said to be preferred to marriage to avoid the costs associated with divorce (Vignoli & Salvini, 2014). Such worry may represent a further selection mechanism—i.e. those who fear the costs of divorce and do transition to marriage would be more likely to choose the separation of property (Hiekel et al., 2014)—a mechanism that we, however, are unable to account for with our data. We therefore formulate a third, competing hypothesis:

#### H3 (Inertia)

Couples with a prior experience of cohabitation will be more likely to opt for separation (vs. community) of property at marriage compared to directly married couples, independently of whether confounders are controlled for or not.

According to H3, we would expect to find a significant difference between the two couple types, both in the null model and in the full model with control variables.

## Data and Methods

We use the Italian multipurpose survey on Family, Social Subjects, and life-cycle, collected in 2016. Each respondent who married at least once was asked about their own and their spouse's socio-demographic characteristics at the time of their first marriage, along with information on the chosen property regime and characteristics of their wedding. Moreover, the survey collects retrospective information on past unions, including premarital cohabitation. Our sample consists of respondents, men, and women, who married at least once, irrespective of whether their first marriage is still intact at the time of interview or had been dissolved. Each respondent is asked to recall own and his/her spouse’s characteristics (age, presence of children, duration of dating period, etc.) at the time of their first marriage. We combine information of the respondent and his/her first spouse (as reported by the respondent) and use the couple as the unit of analysis in our models. We consider opposite-sex marriages only (for same-sex unions, marriages are unavailable in Italy, while civil partnerships exist since 2016).

These survey data represent a unique source of information for studying intra-couple resource pooling in Italy. As we are interested in the comparison between the directly married and those with a premarital cohabitation experience, and as premarital cohabitation is rare in the earlier married cohorts, we base our analyses on marriages celebrated from 1967 onwards (only 41 couples out of 6,264 who married before 1967 experienced premarital cohabitation). In our sample overall 14% of ever-married respondents experienced premarital cohabitation. This share increased across successive marriage cohorts, from less than 5% for the cohorts marrying before 1986 to over 40% for the cohorts marrying between 2007 and 2016.

Logistic regression is used to estimate the probability that a couple chooses the community vs. separation of property at marriage. The key explanatory variable is whether the couple cohabited prior to marriage or not. The hypotheses developed in Sect. 4 are based on the comparison between the empty model, aimed at estimating the main effect (i.e. the association between premarital cohabitation and marital property regime without controls), and the full model, where all controls are included. To this aim, we rely on the KHB method developed by Karlson et al. (2012). The KHB method enables to accurately compare coefficient estimates across two nested logistic regression models (a task that, differently from linear regression, could otherwise not be accomplished in nonlinear probability models because, in this case, the estimated regression coefficients are not comparable across nested models) and, at the same time, it estimates possible mediation effects of the control variables.

As control variables, we include socio-demographic and economic characteristics that we identify as possible confounders of the association between premarital cohabitation and marital property regime (see Sect. 4): marriage cohort; area of residence (North, Centre, South, and Islands); the woman’s age when the couple started dating, using both linear and quadratic terms (the man’s age is not included because highly correlated); the marriage ritual, as a proxy for religiosity (religious vs. civil or mixed, where mixed refers to weddings celebrated among partners belonging to different religions). We also control for the length of the dating period leading to marriage or to the start of the non-marital cohabitation (< 2 years, 2–4 years, 4–6 years, 6–10 years, > 10 years), as a proxy for the level of commitment among partners (the longer the relationship, the higher the level of commitment). We use an unconventional measure based on the dating period because the choice of the marital property regime is made at the time of marriage, hence, for the directly married individuals, differently from previous research on income pooling, we cannot use the time since marriage. We do however include, in a robustness check, an additional control variable measuring the duration of their premarital cohabitation for ‘spousal cohabiters’. We then include two controls measuring the economic resources. First, housing tenure, because if one spouse was a homeowner prior to marriage, he/she may prefer to separate property, similarly to what we expect under the inertia hypothesis. Instead, a joint home purchase is likely associated with pooling (Kan & Laurie, 2014). The variable distinguishes whether the couple lived with parents or parents-in-law, in a property which was built or bought for the couple, made available by parents or parents-in-law (inherited or made available with or without payment of a symbolic rent), made available from one of the spouses (who used to live there before the wedding, whether rented or owner-occupied), whether it was rented or other types. Second, we control for the spouses’ relative education when they started dating (the partner’s education is indirectly obtained as a proxy answer from the respondent), because previous studies showed an educational gradient in the likelihood of choosing the separation of property, and also showed a preference for separation of property in couples where women are more educated than men (Fraboni & Vitali, 2019). We further control for the presence of children, identified by previous literature as associated with pooling in case of children in common, or associated with separation in case of children from previous unions. We further distinguish whether a child in common was born or was conceived prior to the wedding, because we know from previous literature that a birth may trigger joint investments and pooling, while a pregnancy may or may not have the same effect. It may be that the marital commitment triggered by the child in common, linked to a preference for pooling, only manifests after the birth. The variable measures whether the spouses were childless at the time of marriage, had (at least) a child in common born prior to the wedding (i.e. born between the date when the couple started dating and the date of marriage), whether a child was conceived prior to the wedding (i.e. born less than nine months after the date of marriage), or a child was born to one spouse from a previous union (i.e. born before the couple started dating).

Variables are measured retrospectively and refer to own and partner’s characteristics at the time of marriage except for the region of residence, which is measured at the time of interview.

## Results

Of all respondents in our survey who ever married, 63.7% chose the community of property as their preferred marital property regime for their first marriage, 29.2% chose the separation of property and 7.1% of respondents report not knowing or not remembering about their chosen marital property regime (Table 1). As could be expected, this latter percentage is high among earlier marriage cohorts, whose marriage took place many years before the survey interview (e.g. 7.4% among respondents who married between 1967 and 1976), but it is even higher among the most recent marriage cohorts: 8.9% of respondents who married between 2007 and 2016 report not knowing or not remembering their marital property regime. The share of those who do not know about their marital property regime is higher among the younger and lower-educated spouses, those who live with their parents (in law), those in civil/mixed marriages, those with a shorter dating period, and those who had a child born before marriage. Interestingly, across all socio-demographic stratifications, the share of respondents who do not know or do not remember about their marital property regime is generally lower among spouses who previously cohabited, compared to those who directly married (Table 1).

**Table 1:** People ever married by premarital cohabitation experience, marital property regime at first marriage, and other characteristics. Year 2016 (per 100 people ever married)

	Premarital cohabitation								Total			
	No				Yes							
	Marriage regime			Total	Marriage regime			Total	Marriage regime			Total
	Comm	Separ	dk		Comm	Separ	dk		Comm	Separ	dk	
Overall sample	66.9	25.8	7.3	100	43.3	50.6	6.1	100	63.7	29.2	7.1	100
Marriage cohort												
1967–1976	81.6	11.1	7.4	100	70.8	17.7	11.5	100	81.4	11.2	7.4	100
1977–1986	76.6	17.8	5.6	100	68.1	24.6	7.3	100	76.2	18.2	5.6	100
1987–1996	63.7	29.7	6.7	100	50.8	42.6	6.6	100	62.5	30.9	6.7	100
1997–2006	51	40.9	8.2	100	42	52.8	5.3	100	48.9	43.6	7.5	100
2007–2016	44.3	44.9	10.8	100	36.4	57.6	6	100	41.1	50	8.9	100
Female age at first marriage												
Missing	50.4	34.4	15.2	100	46.7	42.1	11.2	100	49.9	35.5	14.6	100
Below 20	76	12.2	11.9	100	80.1	8.6	11.3	100	76.1	12	11.9	100
20–24	73	20.4	6.6	100	57.7	33.1	9.3	100	72	21.2	6.8	100
25–29	58.5	35.9	5.6	100	41.2	52.9	5.9	100	55.9	38.5	5.6	100
30–34	54.4	40.7	4.9	100	33.3	63.1	3.6	100	47.5	48	4.5	100
35–39	51.1	40.5	8.4	100	35	59.3	5.7	100	44.9	47.8	7.4	100
40 and more	50.9	40.9	8.3	100	38.3	57.4	4.3	100	44.1	49.7	6.1	100
Geographical area												
North	63.9	28.6	7.5	100	40.8	53	6.1	100	59.5	33.2	7.3	100
Centre	65.8	26.8	7.4	100	45.3	48.2	6.5	100	62.6	30.1	7.3	100
South and Islands	71	22	7	100	51	43.9	5.2	100	69.8	23.3	6.8	100
Housing Tenure												
Missing	66.8	19.8	13.4	100	54.3	45.7	–	100	63.9	25.8	10.3	100
With parents/in law	66.7	19.5	13.8	100	50.9	36.8	12.3	100	65.4	20.9	13.7	100
Built/bought	64.5	31.1	4.4	100	41.7	56.1	2.3	100	61	34.9	4.1	100
Available from parents/in law	61.5	33.5	5	100	36	59.8	4.2	100	58.4	36.6	4.9	100
Available from one of them	56.1	38.6	5.3	100	31.9	64.6	3.4	100	49.4	45.8	4.8	100
Rented	73.1	20	6.8	100	48	44.1	8	100	69.8	23.2	7	100
Other	56.8	26.5	16.8	100	54.4	30.3	15.3	100	56.3	27.2	16.5	100
Rite of celebration												
Missing	68.9	27.2	3.9	100	39.4	60.6		100	66.9	29.4	3.7	100
Civil/mixed	61.3	27.5	11.2	100	44.3	48.5	7.2	100	56.8	33.1	10.2	100
Religious	68.7	25.2	6.1	100	42.2	53.1	4.7	100	66.4	27.7	6	100
Duration of premarital cohabitation (years)												
Missing					44	42.3	13.6	100	44	42.3	13.6	100
No premarital cohabitation	66.9	25.8	7.3	100					66.9	25.8	7.3	100
Less than 23 months					47	45.8	7.2	100	47	45.8	7.2	100
24–47 months					39.3	55.1	5.5	100	39.3	55.1	5.5	100
More than 48 months					42	53.2	4.8	100	42	53.2	4.8	100
Couple’s education at eng												
Missing	55.1	17.7	27.2	100	66.2	21.8	12.1	100	56.1	18	25.9	100
Both up to compulsory	73.6	17.8	8.6	100	54.6	38.2	7.2	100	72.1	19.4	8.5	100
Both upper secondary	57.2	38.8	4	100	39.1	54.8	6.1	100	53.6	42	4.4	100
Both tertiary	38.9	54.1	7	100	21.5	77.8	0.8	100	32.6	62.6	4.8	100
He is more educated	66.4	28.4	5.2	100	44.2	48.7	7.1	100	63.4	31.1	5.5	100
She is more educated	58.1	36.2	5.7	100	38.5	56.5	5	100	53.4	41.1	5.5	100
Duration of engagement (years)												
Missing	53.7	32.2	14.2	100	50.8	33.4	15.8	100	53.3	32.3	14.4	100
Less than 2 years	67.7	20.7	11.6	100	49.5	41.9	8.7	100	66	22.7	11.3	100
> = 2 & < 4 years	70.9	22.7	6.4	100	42.6	51.2	6.2	100	67.9	25.8	6.4	100
> = 4 & < 6 years	68.6	26.2	5.2	100	47.3	46.1	6.6	100	66.1	28.5	5.3	100
> = 6 & < 10 years	62.7	33.4	3.9	100	40.5	56.5	3	100	58.7	37.5	3.8	100
> = 10 years	56.4	38.1	5.5	100	38.1	55.7	6.3	100	50.4	43.8	5.8	100
Presence of children at the time of first marriage												
No child	66.4	26.7	6.9	100	40.9	53	6.1	100	63.7	29.5	6.8	100
Child in common conceived before marriage	71.4	20.5	8.1	100	47.3	50.1	2.7	100	68.2	24.4	7.4	100
Child in common born before marriage	66	22.7	11.3	100	48.4	44.2	7.4	100	56.8	33.9	9.3	100
Child from previous partner	58.8	26	15.2	100	40.2	51.5	8.3	100	53.3	33.6	13.1	100

**Fig. 1:**
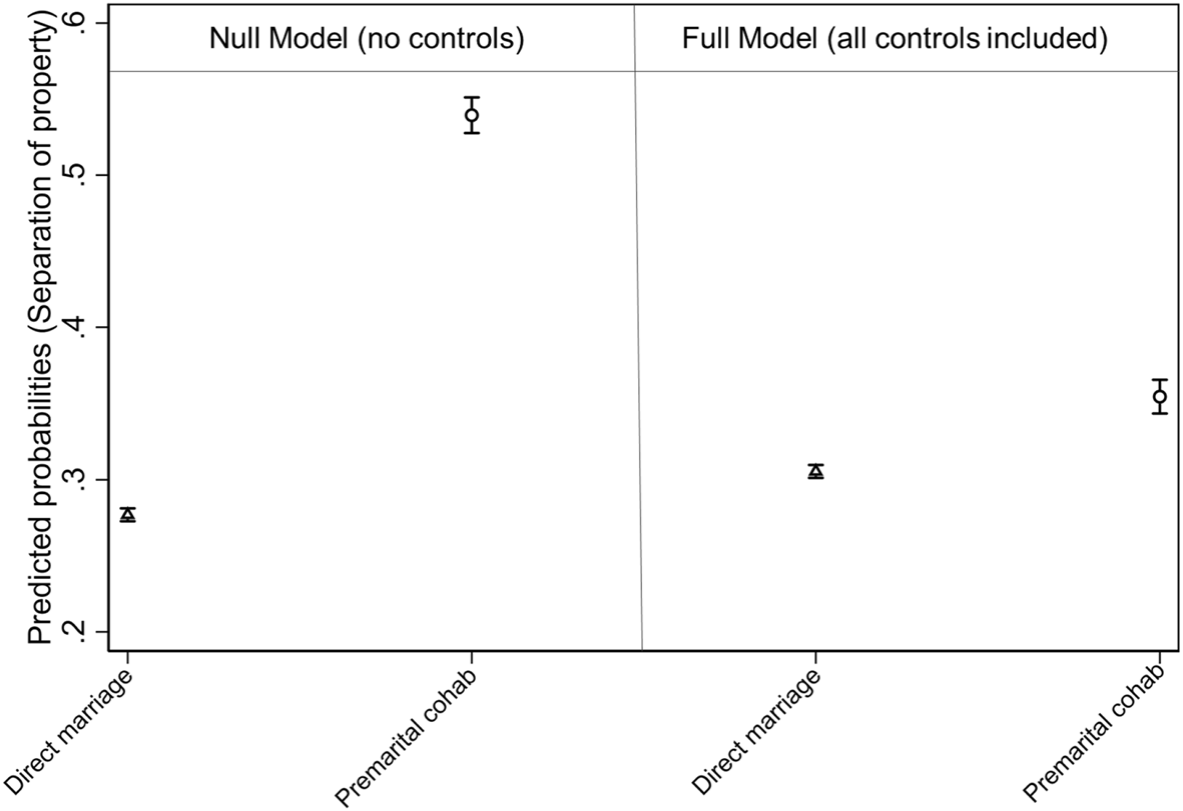
Average adjusted predictions of choosing separation (vs. community) of property regime by premarital cohabitation, with 95% confidence interval *Note*: Predicted probabilities are computed on the basis of model estimates presented in Table 2

In the following analyses, we exclude couples with missing information on the marital property regime (for the equivalent of Table 1 on the restricted sample, see Table A1 in Appendix). In this restricted sample, the separation of property is chosen by 27.8% of directly married couples, against 53.9% among previously cohabiting couples. These differences are statistically significant: the logistic regression model estimates that on average, in the population, the predicted probability of choosing separation of property among directly married individuals ranges between 0.27 and 0.29, while it ranges between 0.52 and 0.56 (i.e. it doubles) among ‘spousal cohabiters’ (Fig. 1, Null Model). Hence, we find descriptive evidence that the way spouses enter into a marriage influences the way they will administer their assets. In a second step, we control for several known confounding factors which may explain selection into cohabitation vs. marriage, and which could influence the chosen marital property regime (Table 2).

**Table 2:** Results from linear logistic models on the probability of choosing separation (vs. community) of property regime, null model, and full model including all control variables

	Null Model				Full Model		
	O.R		S.E		O.R		S.E
Premarital Cohabitation (Ref. NO)							
Yes	3,057	***	0.158		1.300	***	0.084
Marriage cohort (Ref. 2007–2016)							
1967–1976					0.214	***	0.018
1977–1986					0.332	***	0.025
1987–1996					0.568	***	0.039
1997–2006					0.833	**	0.054
Mean age at first marriage, woman					1.139	***	0.021
Mean age at first marriage, woman squared					0.998	***	0.000
Geographical area (Ref. South and Islands)							
North					1.561	***	0.077
Centre					1.299	***	0.079
Rite of celebration (Ref. Religious)							
Civil/mixed					1.013		0.049
Couple’s education at engagement (Ref. Both up to compulsory)							
D.K.					0.961		0.201
Both upper secondary					1.620	***	0.093
Both tertiary					3.219	***	0.430
He is more educated					1.322	***	0.081
She is more educated					1.541	***	0.105
Duration of engagement (Ref. < 2 years)							
> = 2 & < 4 years					0.974		0.059
> = 4 & < 6 years					1.093		0.071
> = 6 & < 10 years					1.283	***	0.082
> = 10 years					1.302	**	0.110
Housing Tenure (Ref. Rented)							
With parents/in law					1.113		0.079
Built/bought					1.211	**	0.067
Available from parents/in law					1.551	***	0.095
Available from one of them					1.590	***	0.139
Other					1.204		0.154
Presence of children at the time of first marriage (Ref. No child)							
Child in common conceived before marriage					0.998		0.067
Child in common born before marriage					0.695	***	0.064
Child from previous partner					0.917		0.157
Constant					0.041	***	0.012
Number of obs.	13,123				13,123		
LR chi2(1)	462.97	LR chi2(27)			2214.3		
Prob > chi2	0.000				0.000		
Log likelihood	− 7788.3772				− 6912.7093		

*p < 0.05; **p < 0.01; ***p < 0.001

Once controls are included in the regression model, the predicted probability of choosing separation of property among directly married individuals ranges between 0.30 and 0.31, while it ranges between 0.33 and 0.38 among ‘spousal cohabiters’ (Fig. 1, Full Model).

The point estimate obtained for spousal cohabiters is sightly higher than the one obtained for directly married couples (the predicted probability of choosing separation vs. community of property equals to 0.35 and 0.31, respectively) and the two 95% confidence intervals do not overlap. On this basis, one may conclude that couples with a premarital experience of cohabitation are significantly more likely to keep their assets separate compared to couples who transition to marriage directly, even after controls, hence after selection mechanisms are accounted for. In other words, one may conclude that regression results show support for H3 (inertia). Looking beyond statistical significance, however, we need to understand to what extent a difference of 0.04 in the predicted probabilities of choosing separation of property among the two couple types is meaningful or not. Statisticians warn that, in large samples such is ours (N. = 13,123), p values will simply be ‘too large to fail’ the null hypothesis of no difference (see, e.g. Lin et al., 2013; Wasserstein et al., 2019). Following the ‘two one-sided test’ (TOST) approach (Rainey, 2014), we reflect on what a meaningful effect could be for the estimated coefficient of the dichotomous variable measuring the experience of a premarital cohabitation (yes/no). In the odds ratio scale, we set the minimal substantively meaningful coefficient equal to 3 (i.e. as in the null model), or 1.5 (i.e. we would consider the behaviour of ‘spousal cohabiters’ to be meaningfully different from the behaviour of directly married couples if the odds of choosing separation of property for ‘spousal cohabiters’ was at least three times or 50% higher, respectively, than the odds for the directly married, controlling for the other variables. Results from the TOST approach fail to reject the null hypothesis of no difference between the two couple types (the 90% confidence interval for the odds ratio ranges between 1.2 and 1.4, hence it does not include 3, nor 1.5). While the choice of these meaningful coefficients is arbitrary, this exercise shows that the original effect found in the null model is considerably reduced after the inclusion of controls.

In a further step, we compare the effect of premarital cohabitation on separation of property in the null and full models, and test for mediation effects of the control variables on the relationship between premarital cohabitation and separation of property using the KHB mediation analysis. As possible confounders for the association between premarital cohabitation and marital property regime, following the review of the literature, we consider all the factors potentially responsible for selection mechanisms which we could measure in our data: marriage cohort, age, geographical region, duration of relationship, presence of children, partners’ education, housing tenure, and religiosity. With the KHB method, we test whether and to what extent controlling for each of these factors reduces the magnitude and significance of the effect of premarital cohabitation on separation of property.

Table 3 shows the results of the mediation analysis. We show the disentangled indirect effect of each confounder based on the full model. Overall, the confounders explain about 80% of the total effect of pre-martial cohabitation on the likelihood of choosing separation of property. This high overall confounding probability suggests that selection mechanisms are indeed in place: the total effect of premarital cohabitation on separation of property is, for a large part, due to other factors associated both with separation of property and with premarital cohabitation, hence lending support to H1 (Selection effects).

**Table 3:** Mediation analysis (KHB) of premarital cohabitation on separation of marital property regime. Percentage explained

	Full model		
	% Explained		s.e
Marriage cohort (Ref. 2007–2016)			
1967–1976	36.48	***	0.026
1977–1986	18.52	***	0.018
1987–1996	4.84	***	0.010
1997–2006	− 2.61	*	0.013
Mean age at first marriage, woman	16.37	***	0.031
Geographical area (Ref. South and Islands)			
North	8.65	***	0.013
Centre	0.67	***	0.004
Rite of celebration (Ref. Religious)			
Civil/mixed	− 0.09		0.018
Couple's education at eng. (Ref. Both up to compulsory)			
Both upper secondary	5.14	***	0.010
Both tertiary	6.36	***	0.013
He is more educated	− 0.31	***	0.003
She is more educated	5.04	***	0.010
Do not know	0.01		0.001
Duration of engagement (Ref. < 2 years)			
> = 2 & < 4 years	0.01		0.005
> = 4 & < 6 years	− 0.5		0.003
> = 6 & < 10 years	2.13	***	0.007
> = 10 years	4.52	**	0.014
Housing Tenure (Ref. Rented)			
With parents/in law	− 0.57		0.005
Built/bought	0.65	**	0.004
Available from parents/in law	− 1.13	***	0.005
Available from one of them	3.05	***	0.009
Other	0.16		0.002
Presence of children at the time of			
first marriage (Ref. No child)			
Child in common conceived before marriage	0		0.000
Child in common born before marriage	− 7.16	**	0.023
Child from previous partner	− 0.24		0.004
All mediators	79.50	***	
Number of obs	13,123		
Pseudo R2	0.14		

The percentage explained is the disentangled effect when all variables are simultaneously included in the Full Model

*p < 0.05; **p < 0.01; ***p < 0.001

**Fig. 2:**
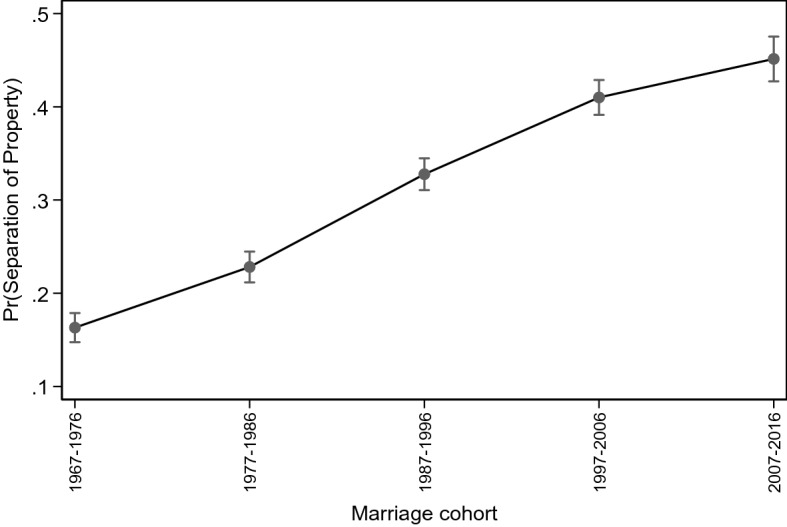
Average adjusted predictions of choosing separation (vs. community) of property regime by marriage cohort, with 95% confidence interval

Marriage cohort is the most influential confounder of all: belonging to the cohorts 1967–1976 and 1977–1986 accounts for 36% and 19%, respectively, of the effect of premarital cohabitation, while belonging to more recent marriage cohorts has a considerably smaller selection effect. These results suggest that those who cohabited prior to marriage during the 1960s-1980s, i.e. in a period when cohabitation was rare in Italy, were a highly selected group who were also considerably more likely to opt for separation of property compared to the directly married. Such selection mechanism gradually loses importance across successive marriage cohorts, as both cohabitation and separation of property become common. Figure 2 shows how the predicted probability of choosing the separation of property obtained from the full model increases considerably across successive marriage cohorts from the late 1970s to the mid-2000s, then continues increasing at a slower pace (Fig. 2). Overall, separation of marital property is becoming the preferred arrangement among the most recent marriage cohorts—a preference which is more visible among the ‘spousal cohabiters’, but that is growing also among the directly married (Table 1).

Other selection mechanisms are found for woman’s age (the older the woman, the higher the likelihood of both cohabitation and separation of property), geographical region (couples residing in Northern Italy are more likely to both experience cohabitation and choose separation of property), partners’ relative education (higher-educated spouses as well as couples where she is more educated than him have a higher likelihood of both cohabiting and separating property; instead the effect of when he is more educated than her is suppressive, because the likelihood of cohabiting is lower while the community of property is more likely), duration of engagement (the longer the duration, the higher the likelihood of both cohabitation and separation of property) and housing tenure (if a property is available to one of the spouses, both cohabitation and separation are more likely). Instead, having a (common) child born prior to the marriage and, to a lesser extent, having a child from a previous union, have a suppressing effect on the association between cohabitation and separation of property, because both circumstances lower the likelihood of choosing separation vs. community (see Table 2). The rite of celebration and the wife being pregnant have no mediation effect.

Results concerning the sign of control variables in the full model are as expected (see Table 2 and Figures S1–S6 reporting predicted probabilities computed for values assumed by control variables in the supplementary material). The separation of property is most likely when both spouses have a high educational attainment, it is lowest when they both have low educational attainment and it is intermediate when both are medium-educated or have different educational attainments. As far as housing tenure is concerned, the probability of choosing the separation of property is highest for couples that, upon marriage, live in a property which was available to one spouse before the start of the spousal co-residence, an owner-occupied property acquired via inheritance by one of the spouses, or available from one’s parents. If the couple had a child who was born prior to the marriage, the couple is less likely to choose the separation of property compared to childless couples. But this result does not apply when the bride is pregnant at the time of marriage, nor—as expected from previous literature—if the child was born from a previous union (i.e. before the couple started dating). The duration of the relationship, measured by the length of the dating period preceding marriage or cohabitation, does not appear to matter for the chosen marital property regime. The probability of choosing the separation of property is highest in the North of Italy, intermediate in the Centre and lowest in the South and Islands and it increases with the bride’s age, but in a nonlinear way (Table 2).

In a robustness check (Model 2 in Figure A2), we add a control for the duration of non-marital cohabitation for ‘spousal cohabiters’, coded as 0 for the directly married. Results show no clear association between duration of premarital cohabitation and chosen marital property regime. Only for couples cohabiting between 24 and 47 months do we find a significantly higher likelihood of choosing the separation vs. community of property at marriage compared to directly married individuals. In accordance with previous literature, once other relevant confounders are controlled for, we find no difference in the chosen marital property regime between directly married couples and couples with a long history of premarital cohabitation. However, contrary to previous findings, we also find a similar behaviour in resource pooling between cohabiting couples with a short cohabitation history of less than two years and directly married couples.

## Limitations

Our study is not without limitations. First, whereas for the respondents we have full information on all socio-demographic variables at the time of interview and at the time of first marriage, for their (first) spouse this is not always the case, unless first marriages remain intact until the time of interview. In our operationalization, this issue is overcome by indirectly obtaining the spouse’s characteristics via proxy answers from the respondent. In this way, we can include in the analyses all first marriages, including those that dissolved. Nevertheless, this approach prevents us from including other control variables measuring additional characteristics of the spouse: for respondents who are widowed, divorced, separated, or re-married at the time of interview, the previous spouse is, by definition, no longer a member of the family and the respondent is not asked, e.g. about his/her first spouse’s marital status at the time of marriage, etc. Furthermore, for these individuals, questions about their first (dissolved) marriage may be intrusive and responses to the survey questions may be influenced by this intrusion.

We also acknowledge that some of our variables may be imprecisely constructed, due to data limitation. One such case is the variable measuring presence of children at the time of marriage, distinguishing between children in common born vs. conceived before marriage vs. children born before marriage from a previous relationship. This variable is computed combining information on age at childbirth, computed from the children’s date of birth and the self-reported age when the couple started dating. While children’s date of birth is a precise measure, age at dating may be affected by recall errors and is less precise. Information referring to the partner’s age when they started dating and relative educational level is indirectly obtained as proxy answer from the respondent and this too could be in part affected by measurement errors, especially in the case of disrupted marriages. We further acknowledge possible difficulties of recalling information of the wedding’s characteristics for older marriage cohorts. The variable measuring the geographical region is measured at the time of interview, which may differ from the region where the marriage was celebrated or where the couple lived upon marriage. The control variable for the wedding ritual (religious vs. civil) may not capture religiosity, but rather tradition and preference for a more spectacular church wedding (Vignoli & Salvini, 2014).

Finally, we acknowledge that, for lack of data, this paper: 1) does not take fully into account possible gender differences in the accumulation of wealth during marriage; 2) it does not consider whether the choice of the marital property regime is influenced by the intra-couple distribution of paid and care work at the time of marriage; 3) it does not consider whether there is an association between gender of the parent who had a child from a previous union and likelihood of choosing separation of property. For a discussion on a gendered perspective on who, between the woman and the man, according to their economic characteristics may have more power in choosing their marital property regime in Italy see Fraboni and Vitali (2019).

## Conclusions

Previous research has shown a ‘cohabitation–marriage gap’ in resource management and pooling, with cohabiting couples being more likely to keep at least part of their economic resources separate compared to married couples (Evans & Gray, 2021; Hamplova & Bourdais, 2009; Lyngstad et al., 2011; Winkler, 1997). We add to this literature by studying differences in (one aspect of) resource pooling among couples who directly marry and those who marry after a previous experience of non-marital cohabitation. Such a comparison offers a litmus test of theories of marriage in relation to ‘wealth in couples’ and with regard to couples’ resource management and pooling.

This paper focuses on a comparison between directly married and previously cohabiting spouses in relation to one aspect of resource pooling: the marital property regime, regulating the legal ownership of assets accumulated during marriage and in case of divorce in Italy. Italy is an interesting case study as, due to data limitations, so far we know little about couples’ resource management and pooling for Italy.

Results show a significant legacy of prior experience of cohabitation with regard to the decision about how marital wealth should be distributed: a prior experience of cohabitation among two spouses increases the likelihood of choosing the separation vs. community of property compared to directly married couples. However, once relevant confounders are accounted for, the association between premarital cohabitation and the choice of marital property regime becomes much weaker. Our data, the 2016 Italian multipurpose survey on ‘Family, social subjects and life-cycle’, provides unique information on the characteristics of spouses at first marriage. These rich data enabled us to control for known selection mechanisms (i.e. factors typically associated with cohabitation vs. direct marriage and with resource pooling) identified by previous research (i.e. length of relationship, presence of common and step-children, religiosity and other socio-demographic and economic confounders). Mediation analyses performed via the KHB method show that such socio-demographic and economic confounders mediate the association. Overall, confounders (i.e. selection effects) explain about 80% of the effect of premarital cohabitation on separation of property. In other words, it is not the experience of premarital cohabitation per se that has an effect on how spouses allocate the ownership of their wealth. Rather, a set of confounders drives a selected group of couples into premarital cohabitation instead of direct marriage, and this same set of confounders drives spouses into separation instead of community of property. Our results hence support that differences in resource management and pooling between cohabiters and married individuals observed in previous literature, are due to selection mechanisms making the two groups essentially different (Hamplová et al., 2014; Lyngstad et al., 2011).

Our results cast doubt on the argument that (currently) cohabiting partners opt more often for separation of resources than married people because non-marital cohabitation lacks the legal protection typical of marriage, and therefore discourages partners from making joint investments (Winkler, 1997). Indeed, we find that a prior experience of cohabitation among spouses remains associated with resource separation, even upon marriage, i.e. when spouses benefit from the legal protection for joint investments offered by the marital contract—if selection mechanisms are not appropriately controlled for (i.e. in the null model, when relevant confounders are not included). Our results are compatible with Hiekel et al. (2014) who find that, after relevant controls are considered, income separation is equally likely among ‘spousal cohabiters’ and directly married couples in four out of six countries analysed, among which is Germany, the country closer to Italy in terms of family patterns and gender equality, among those included in their sample.

Other previous studies concluded that the observed differences must be due to the intrinsically different nature of marriage and cohabitation, i.e. different levels of commitment, different normative scripts related to resource pooling, and different legal protection (Hiekel et al., 2014; Lyngstad et al., 2011). This conclusion however cannot explain why differences in resource pooling remain significant when comparing ‘spousal cohabitations’ and direct marriages: both couple types choose their marital property regime at the time of marriage, i.e. when the spouses commit to a long-term relationship, start sharing marriage-related social expectations and are offered legal protection for their future joint investments.

Our study shows that most of the differences in the choice of marital property regime between ‘spousal cohabiters’ and directly married couples can be explained by selection mechanisms. Nevertheless, a modest statistically significant effect remains. This remaining effect could be the result of selection not accounted for by the control variables in this study or it could be the result of what we called ‘the inertia hypothesis’, i.e. of the fact that spouses who cohabited prior to the marriage may be accustomed to sharing some economic resources but are likely to keep at least some of their economic resources separately, as shown by empirical research reviewed in this paper. Upon marriage, ‘spousal cohabiters’ may be more likely than directly married spouses to choose separation vs. community of property, all else being equal, due to this previous experience of holding at least some separate resources.

Results in this paper are based on the marital property regime, a unique measure of wealth—rather than income—pooling. The marital property regime has the status of law, rather than being a practice of resource management in everyday life, generally self-reported by one couple member. Hence, while income pooling and resource management practices can be changed at any point during the marital relation, the choice of the marital property regime is made at a precise point in time, i.e. at the beginning of the contractual married life, and has important implications for the ownership and distribution of wealth among couples during marriage and in case of divorce. Wealth ownership measures one specific aspect of resource pooling, and future studies should compare directly married and previously cohabiting spouses according to different measures of income sharing and everyday practices of resource allocation.

Beyond the main focus on the association between premarital cohabitation and marital property regime, our study also brings to light five aspects which may be of interest to scholars researching resource pooling among partners. First, to our knowledge, this is the first study to apply KHB mediation analysis to address the existence of selection mechanisms driving (premarital) cohabitation and resource pooling. These methods can be fruitfully applied in future studies of resource management and pooling because they allow disentangling the role of selection mechanisms in explaining the effect of (pre-martial) cohabitation on the evaluation of the ‘cohabitation–marriage gap’. KHB also enables to identify which factors are responsible for mediating the association between (premarital) cohabitation and propensity to separate economic resources. Earlier literature on resource management and pooling has not properly taken selection into account, despite relying on selection mechanisms as an explanation for the cohabitation–marriage gap.

Second, we find evidence that spouses prefer to keep their assets separate when they are more likely to already own assets in their name at the time of marriage. For instance, the separation of marital property is preferred when one spouse already has a housing property purchased, inherited, or otherwise available to them prior to marriage. Our results therefore confirm Kan and Laurie’s (2014) findings that sole ownership of housing is a strong predictor of separate savings, investments, and debts. Similarly, the likelihood of choosing separation of property increases with the spouses’ educational level. Furthermore, we find that when the woman is more educated than her spouse, the likelihood of choosing the separation of property is higher compared to when the man is more educated, although such difference is not statistically significant once controlling for other confounders. This result confirms previous findings that women’s economic advantage compared to their spouses is related to separation of resources (Burgoyne, 1990; Lott, 2017; Pepin, 2019), including separation of marital property (Fraboni & Vitali, 2019).

Third, contrary to what is found in previous literature, we find that the likelihood of choosing separation of property at marriage is the same among childless couples, couples with a child born before they started dating—a proxy for children born from a previous union—and couples who conceived a child before the wedding. Only for couples with a child in common born prior to the wedding do we find a lower likelihood of choosing separation of property, compared to childless couples and couples who conceived a child before the wedding. From previous international research, we know that having a child from a previous union is associated with separation of property. For Italy, this result does not seem to hold. We acknowledge, however, that our measure of children conceived before marriage may be imperfectly computed (see Sect. 6). We also acknowledge that the number of couples with a stepchild is small in our sample, hence the related confidence intervals are wide. And yet, the point estimate is lower—not higher, as one would expect—than the point estimate for childless couples. This estimate is closer to, and not statistically different from, the estimate for couples with a common biological child born before the wedding. These results suggest that, at least in the Italian context, experiencing childbirth, as well as living with a child, even if not a common biological child to both partners, is positively related to pooling marital property. Instead, conception per se, i.e. the idea or anticipation of becoming a parent, is irrelevant to the property regime chosen at the time of marriage.

Fourth, selection mechanisms can change across cohorts and future studies should consider this when pooling together data from multiple cohorts. Our study considers marriage cohorts spanning between 1967 and 2016. Results from the KHB mediation analysis suggest that belonging to earlier marriage cohorts proves to be the most important confounder for the effect of premarital cohabitation on the chosen property regime, meaning that individuals who cohabited prior to marriage in the 1960s, 70 s, and 80 s—i.e. when cohabitation was rare—are a selected group who are also more inclined to separate their marital property. Among more recent cohorts, instead, such a selection mechanism does not hold any longer, because both the experience of cohabitation and the choice of separation of property become commonplace in the population.

Finally, we find that a fairly high share of respondents reports not knowing or not remembering whether they chose the separation vs. community of property at marriage. Almost one in ten respondents whose marriage was celebrated between 2007 and 2016 do not know or do not remember their chosen marital property regime. This is the highest percentage of the sample, followed by that observed for marriages celebrated between 1997 and 2006: worryingly, the more recent the marriage, the higher the share of respondents not knowing about the legal arrangements regulating their marital wealth. This share is lower among spouses who cohabited prior to marriage, compared to those who married directly, across ages, marriage cohort, socio-economic backgrounds, and regions. This result suggests that ‘spousal cohabiters’ are more informed than directly married spouses about the ownership of their wealth and the way their wealth will be shared with their spouse in case of divorce.

### Supplementary Information

Below is the link to the electronic supplementary material.

Supplementary file1 (DOCX 91 kb)

## Appendix

See Tables

**Table 4:** People ever married by premarital cohabitation experience, marriage regime at first marriage, and other characteristics, excluding respondents with missing values on the marital property regime. Year 2016 (per 100 people ever married)

	Premarital cohabitation						Total		
	No			Yes					
	Marriage regime		Total	Marriage regime		Total	Marriage regime		Total
	Comm	Separ		Comm	Separ		Comm	Separ	
Overall sample	72.2	27.8	100	46.1	53.9	100	68.6	31.4	100
Marriage cohort									
1967–1976	88	12	100	80	20	100	87.9	12.1	100
1977–1986	81.1	18.9	100	73.5	26.5	100	80.7	19.3	100
1987–1996	68.2	31.8	100	54.4	45.6	100	66.9	33.1	100
1997–2006	55.5	44.5	100	44.3	55.7	100	52.9	47.1	100
2007–2016	49.6	50.4	100	38.7	61.3	100	45.2	54.8	100
Female age at first marriage									
Missing	59.5	40.5	100	52.6	47.4	100	58.5	41.5	100
Below 20	86.2	13.8	100	90.3	9.7	100	86.4	13.6	100
20–24	78.2	21.8	100	63.6	36.4	100	77.3	22.7	100
25–29	62	38	100	43.8	56.2	100	59.2	40.8	100
30–34	57.2	42.8	100	34.5	65.5	100	49.7	50.3	100
35–39	55.8	44.2	100	37.2	62.8	100	48.4	51.6	100
40 and more	55.5	44.5	100	40.1	59.9	100	47	53	100
Geographical area									
North	69.1	30.9	100	43.5	56.5	100	64.2	35.8	100
Centre	71.1	28.9	100	48.5	51.5	100	67.6	32.4	100
South and Islands	76.3	23.7	100	53.8	46.2	100	74.9	25.1	100
Housing Tenure									
Missing	77.1	22.9	100	54.3	45.7	100	71.2	28.8	100
With parents/in law	77.4	22.6	100	58	42	100	75.8	24.2	100
Built/bought	67.5	32.5	100	42.6	57.4	100	63.6	36.4	100
Available from parents/in law	64.7	35.3	100	37.6	62.4	100	61.5	38.5	100
Available from one of them	59.2	40.8	100	33.1	66.9	100	51.9	48.1	100
Rented	78.5	21.5	100	52.1	47.9	100	75	25	100
Other	68.2	31.8	100	64.2	35.8	100	67.5	32.5	100
Rite of celebration									
Missing	71.7	28.3	100	39.4	60.6	100	69.5	30.5	100
Civil/mixed	69.1	30.9	100	47.8	52.2	100	63.2	36.8	100
Religious	73.2	26.8	100	44.3	55.7	100	70.6	29.4	100
Duration of premarital cohabitation (years)									
Missing				51	49	100	51	49	100
No premarital cohabitation	72.2	27.8	100				72.2	27.8	100
Less than 23 months				50.7	49.3	100	50.7	49.3	100
24–47 months				41.6	58.4	100	41.6	58.4	100
More than 48 months				44.1	55.9	100	44.1	55.9	100
Couple's education at eng									
Missing	75.7	24.3	100	75.3	24.7	100	75.7	24.3	100
Both up to compulsory	80.6	19.4	100	58.8	41.2	100	78.8	21.2	100
Both upper secondary	59.6	40.4	100	41.6	58.4	100	56.1	43.9	100
Both tertiary	41.8	58.2	100	21.6	78.4	100	34.2	65.8	100
He is more educated	70.1	29.9	100	47.6	52.4	100	67.1	32.9	100
She is more educated	61.6	38.4	100	40.5	59.5	100	56.5	43.5	100
Duration of engagement (years)									
Missing	62.5	37.5	100	60.3	39.7	100	62.2	37.8	100
Less than 2 years	76.6	23.4	100	54.2	45.8	100	74.4	25.6	100
> = 2 & < 4 years	75.7	24.3	100	45.4	54.6	100	72.5	27.5	100
> = 4 & < 6 years	72.3	27.7	100	50.7	49.3	100	69.9	30.1	100
> = 6 & < 10 years	65.3	34.7	100	41.8	58.2	100	61	39	100
> = 10 years	59.7	40.3	100	40.6	59.4	100	53.5	46.5	100
Presence of children at the time of first marriage									
No child	71.3	28.7	100	43.6	56.4	100	68.3	31.7	100
Child in common conceived before marriage	77.7	22.3	100	48.6	51.4	100	73.6	26.4	100
Child in common born before marriage	74.4	25.6	100	52.2	47.8	100	62.6	37.4	100
Child from previous partner	69.4	30.6	100	43.8	56.2	100	61.4	38.6	100

^4^,

**Table 5:** Results from linear logistic models on the probability of choosing separation (vs. community) of property regime, Robustness check including duration of cohabitation for previously cohabiting couples

	O.R		s.e
Marriage cohort (Ref. 2007–2016)			
1967–1976	0.215	***	0.018
1977–1986	0.335	***	0.025
1987–1996	0.57	***	0.039
1997–2006	0.837	***	0.054
Mean age at first marriage, woman	1.143	***	0.022
Mean age at first marriage, woman squared	0.998	***	0.000
Geographical area (Ref. South and Islands)			
North	1.562	***	0.078
Centre	1.297	***	0.078
Duration of premarital cohabitation (Ref. No premarital cohabitation)			
Less than 23 months	1.214	**	0.106
24–47 months	1.547	***	0.166
More than 48 months	1.217	*	0.128
Rite of celebration (Ref. Religious)			
Civil/mixed	1.018		0.049
Couple's education at engagement (Ref. Both up to compulsory)			
Missing	0.965		0.202
Both upper secondary	1.62	***	0.093
Both tertiary	3.21	***	0.430
He is more educated	1.33	***	0.081
She is more educated	1.54	***	0.105
Duration of engagement (Ref. < 2 years)			
> = 2 & < 4 years	0.965		0.059
> = 4 & < 6 years	1.081		0.071
> = 6 & < 10 years	1.276	***	0.083
> = 10 years	1.307	***	0.113
Housing Tenure (Ref. Rented)			
With parents/in law	1.12		0.080
Built/bought	1.213	***	0.067
Available from parents/in law	1.546	***	0.095
Available from one of them	1.574	***	0.138
Other	1.209		0.155
Presence of children at the time of first marriage (Ref. No child)			
Child in common conceived before marriage	1.002		0.068
Child in common born before marriage	0.709	***	0.068
Child from previous partner	0.925		0.159
Constant	0.039	***	0.012
Number of obs	13,108		
LR chi2(27)	2211.96		
Prob > chi2	0		
Log likelihood	− 6901.462		
Pseudo R2	0.138		

**p* < 0.05; ***p* < 0.01; ****p* < 0.001

^5^
