# Supplementary material for: Pooling of Wealth in Marriage: The Role of Premarital Cohabitation
Source: Eur J Popul. 2022 Aug 9;38(4):721–54. doi: 10.1007/s10680-022-09627-2 (PMC9550889; doi:10.1007/s10680-022-09627-2)
Supplement: Supplementary file 1 — Supplementary file1 (DOCX 91 kb) [file 10680_2022_9627_MOESM1_ESM.docx]

Online Supplementary Material

Figure S1: Average adjusted predictions of choosing separation (vs. community) of property regime by partner’s education, with 95% confidence interval

Figure S2: Average adjusted predictions of choosing separation (vs. community) of property regime by housing tenure, with 95% confidence interval

Figure S3: Average adjusted predictions of choosing separation (vs. community) of property regime by children, with 95% confidence interval

Figure S4: Average adjusted predictions of choosing separation (vs. community) of property regime by dating duration, with 95% confidence interval

Figure S5: Average adjusted predictions of choosing separation (vs. community) of property regime by duration of premarital cohabitation, with 95% confidence interval

Figure S6: Average adjusted predictions of choosing separation (vs. community) of property regime by parental education, by gender of respondent, with 95% confidence interval

| Men | Women |
| --- | --- |
| 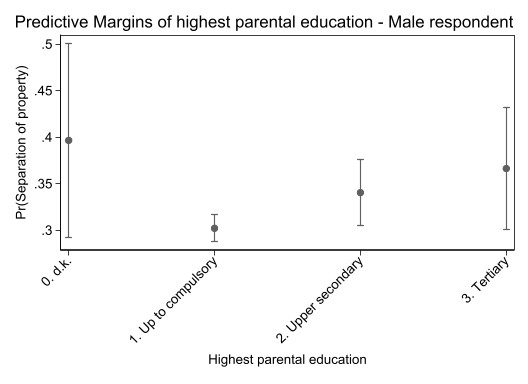 | 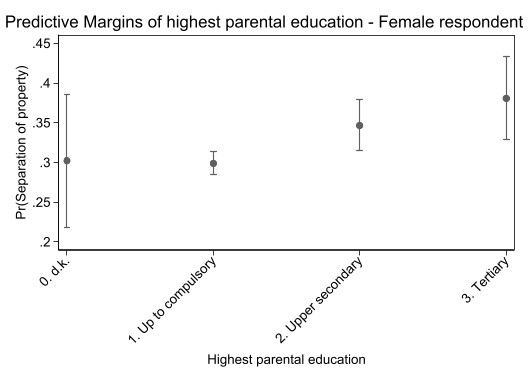 |
